# Engineering macrophages for cancer immunotherapy: emerging insights and therapeutic potential

**DOI:** 10.1038/s44385-026-00072-y

**Published:** 2026-03-02

**Authors:** Anton Block, Xiaoya Liu, Daniel Zhang, Kolade Adebowale

**Affiliations:** 1 Shu Chien-Gene Lay Department of Bioengineering, University of California, San Diego, La Jolla, CA USA; 2 Program in Immunology, University of California, San Diego, La Jolla, CA USA; 3 Moores Cancer Center, University of California, San Diego, La Jolla, CA USA

**Keywords:** Biotechnology, Cancer, Cell biology, Immunology

## Abstract

Macrophages strongly influence cancer progression through their adaptable phenotypes and responses to mechanical and biochemical cues. Their abundance across tumors and links to poor outcomes drive interest in macrophage-targeted therapies. This review highlights macrophage mechanobiology, key behaviors, and the potential of engineered macrophages, using genetic (CAR) or non-genetic “Trojan horse” and “backpacking” strategies, to deliver therapies and reshape the immunosuppressive tumor microenvironment.

## Introduction

**Fig. 1:**
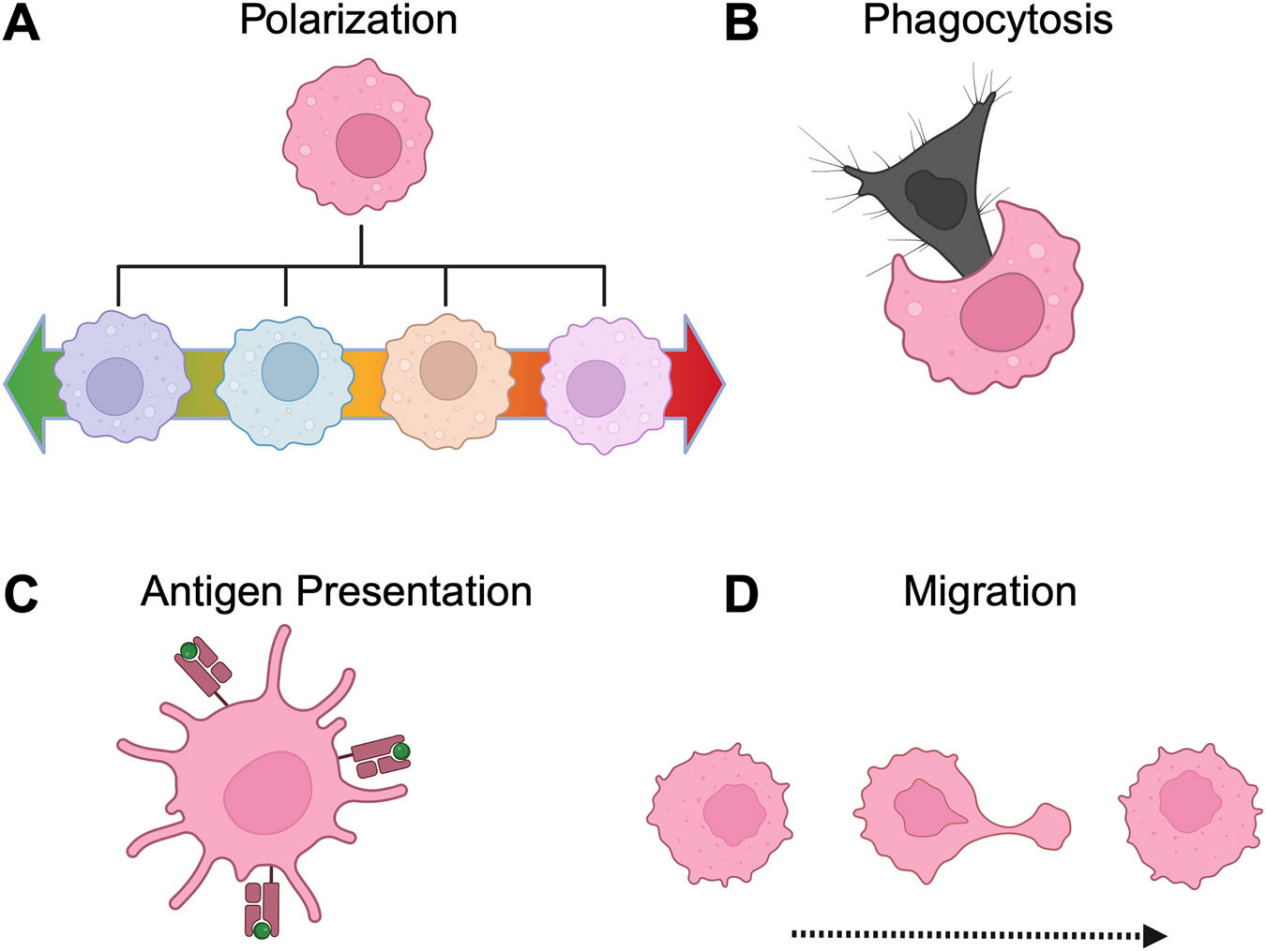
Macrophage plasticity and examples of diverse immune functions. **A** Naïve macrophages in the tumor microenvironment are phenotypically plastic and exist on a pro-inflammatory to anti-inflammatory spectrum. **B** Macrophage (pink) phagocytosing a cancer cell (black). **C** Macrophages can present antigens (green circles) on major histocompatibility complex class 1 (MHC-1) molecules (pink) to CD8^+^ cells. **D** Macrophages can migrate during tissue immunosurveillance. Created in BioRender. Block, A. (2025) https://BioRender.com/1h8p9tz. Cell adhesion and integrins modulate pro-inflammatory and cytokine signaling transduction pathways in monocytes and macrophages^131–133^.

Macrophages play crucial roles in health and disease^1^. They can be tissue-resident or derived from circulating monocytes. Circulating monocytes differentiate into macrophages in response to inflammatory cues released from solid tissues^2,3^. Upon recruitment to tissues, macrophages can adopt diverse phenotypic states in response to the local milieu (Fig. 1), enabling them to participate in many physiological processes, including fibrosis, tissue repair, regeneration, and cancer progression^4–15^. Macrophage phenotypic plasticity has historically been classified into two extremes: an M1-like (pro-inflammatory or anti-tumor) and an M2-like (anti-inflammatory or pro-tumor). These phenotypes are obtained ex vivo by culturing naïve macrophages using diverse protocols, for example, with LPS (and/or IFN-γ, TNF-α, etc.) or IL-4 (or IL-10, IL-13, TGF-β, etc.) respectively^16,17^. Mounting evidence has demonstrated that macrophages exist in phenotypic states that extend beyond the M1/M2 paradigm (anti-tumor/pro-tumor)^18,19^. More recent in vivo characterization has revealed that there is substantial overlap between the gene signatures of ex vivo polarized M1-like and M2-like phenotypes, thereby questioning the physiological relevance of the M1/M2 binary classification^20^. In addition, new evidence suggests that the in vivo functional behavior of macrophages is not always consistent with the M1/M2 paradigm. For instance, recent studies demonstrated that the conventionally defined pro-tumor CD206+ macrophages differentially attracted and activated tumor antigen-specific CD8 T cells to exert an anti-tumor effect^21^. In addition, 60% of the in vivo upregulated genes in tumor-associated macrophages (TAMs) found in brain tumors do not overlap with the M1/M2 genes^19^. TAMs are macrophages within the tumor microenvironment that support or suppress tumor growth and are thought to mostly originate from circulating monocytes. Moreover, even within TAMs, single-cell ‘omics has identified seven TAM sub-populations^16^. It is unclear how these TAM sub-populations evolve, whether they are terminal states, or, more importantly, if they promote or inhibit cancer progression. In some cases, the presence of TAMs is associated with unfavorable cancer outcomes. However, other studies find that TAMs correlate with better outcomes in human colorectal cancer liver metastases, mouse melanoma, and mouse lymphoma^21–23^. Moreover, advances in spatial ‘omics technologies have revealed that it is not simply the presence of macrophages, but their spatial arrangement and cellular associations that determine their functions^24–26^. Together, these findings suggest that macrophage functions are diverse and context-dependent. Therefore, it is crucial to understand the dynamics and functions of macrophage subtypes to enable rational macrophage-targeting therapeutic strategies.

Once in solid tissues, macrophages’ functional behaviors include phagocytosing microbes or dead cells (efferocytosis), modulating their phenotype, presenting antigens to T cells, and migrating through the extracellular matrix (ECM)^27–30^ (Fig. 1). These unique characteristics make them attractive candidates to target desired tissues and perform specific functions in the body. The inflammatory cues that recruit macrophages can result from macrophage detection of pathogens and subsequent cytokine secretion or release of inflammatory factors by cancer. During a pathogenic response, macrophages use pattern recognition receptors to detect pathogen-derived molecules known as pathogen-associated molecular patterns, such as lipopolysaccharide in the bacterial wall, which triggers the activation of macrophages^31–33^. Activated macrophages exhibit high phagocytic activity, leading to pathogen clearance and cellular debris. Furthermore, they can bridge innate and adaptive immunity by recruiting T cells and presenting antigens to activate T cells. In the context of cancer, macrophages are recruited due to tumor-secreted biochemical cues such as macrophage colony-stimulating factor 1 (CSF-1) and monocyte chemoattractant protein-1 (MCP-1), and vascular endothelial growth factor (VEGF)^34^. Once in the tumor, macrophages exhibit phenotypic plasticity, rapidly changing their function in response to the local tumor microenvironment (TME), the complex network of cellular and non-cellular components in a tumor that shapes tumor growth, progression, and therapy response.

## Emerging insights into macrophage behaviors

### Macrophage mechanobiology

Major advances in the past few years have demonstrated that immune cells sense and respond to mechanical and biophysical cues^35–61^. The emerging field of immune cell mechanobiology or mechanoimmunology seeks to identify the molecular mechanisms that govern how biophysical and mechanical cues modulate immune cell functions^62–70^. Indeed, growing evidence suggests that several immunoreceptors, including the T cell receptor (TCR), integrins, PD-L1, PD-L2, Piezo1, are mechanosensitive^55,71–76^. Of these, Piezo1 has been well studied, whereby, Piezo1 signaling has been shown to promote a pro-inflammatory macrophage phenotype through the NFκB and STAT6 pathways^76^. Macrophages in particular are one of the first immune cells found in the developing embryo, which suggests they might be able to sense and respond to diverse cues in tissues^77^. In addition, they are found in most tissues during normal and pathological conditions. Due to their intimate involvement in maintaining tissue homeostasis, they encounter a variety of distinct mechanical and biophysical cues as they migrate through the ECM, move across other cells, communicate with other cells, or experience fluid flow in interstitial spaces, lymph nodes, or blood vessels. Accordingly, macrophages have evolved several strategies to probe their mechanical environment. There are macrophage-sensing strategies, including plasma membrane curvature-sensing proteins and the Piezo1 receptor^76,78^. Increasing evidence has implicated Piezo1 in regulating macrophage functions.

The Piezo1 receptor is part of a family of evolutionarily conserved, nonselective cation channels involved in mechanosensory transduction and has recently been implicated in a wide range of biological processes^79^. The bidirectional crosstalk between Piezo1 and the cytoskeleton suggests it might be important for many biological processes related to mechanotransduction^80,81^. Piezo1 regulates cell-matrix and cell-cell interactions in macrophages. For instance, macrophages regulate Piezo1 activity to modulate activation in response to matrix stiffness^76^. In addition, cyclic hydrostatic pressure activates Piezo1, leading to the initiation of an inflammatory response in mice^82^. Moreover, Piezo1 has been shown to be important for phagocytosis and engulfing apoptotic cells (efferocytosis)^55,83^. The role of mechanosensation is not obvious, but one can imagine that, being professional phagocytes, macrophages must contact, surround, and internalize their targets. Each of these steps will likely involve sensing and producing physical forces by the macrophages. The increase in stiffness of the bacterial cell wall, aged red blood cells, or cancer cells could serve as a mechanical checkpoint to trigger phagocytosis^84–87^. Furthermore, a recent study showed that macrophages can trigger fibroblast activation through Piezo1^75^. A model was proposed whereby macrophages use a_v_β_3_ integrins to pull on fibroblasts, leading to stretching of Piezo1 on the fibroblast, and subsequent calcium influx^75^. This finding suggests that the biophysics of cell-cell contact could play an important role in regulating cellular functions. In sum, accumulating evidence suggests that mechanical and biophysical cues could play major roles in dictating macrophage-driven inflammatory response.

In addition, macrophage phenotype is increasingly recognized as highly sensitive to the mechanical properties of the surrounding extracellular environment, and biomaterials studies have provided key insight into this mechanoregulation^88^. Substrate stiffness can bias macrophage polarization, where softer matrices ( ~ 1-10 kPa) tend to promote M2-like, pro-repair phenotypes, whereas stiffer environments ( ≥ 100 kPa) often enhance M1-like, pro-inflammatory activation through YAP^89,90^. Moreover, Piezo1, which is upstream of YAP, regulates macrophage phenotype: increased stiffness upregulates Piezo1, driving YAP nuclear localization and M1 polarization, while inhibiting YAP can repolarize macrophages toward M2^89,91^. In addition, as substrate stiffness increases, macrophages exhibit enhanced spreading, stress fiber formation, and maturation of adhesions, reflecting cytoskeletal adaptation to higher mechanical load^90,92^. Together, these findings demonstrate that macrophages integrate biophysical cues with classical biochemical stimuli in their local microenvironment to fine-tune their phenotype and function.

### Phagocytosis and antigen presentation

Macrophages are the professional phagocytes of the immune system and are capable of phagocytosing red blood cells, microbes, amyloid-β fibrils, or cancer cells^55,93–96^. Macrophage engulfment of cancer cells is a promising anticancer treatment because it eliminates cancer cells, leading to the suppression of tumor growth^95^. Accordingly, multiple lines of evidence have demonstrated that several cancers have evolved mechanisms to evade engulfment by macrophages. Specifically, it is thought that the CD47-SIRPα signaling axis is an important mediator of phagocytosis^97^. Cancer cells often overexpress the CD47 (“don’t eat me”) ligand, which triggers the inhibitory SIRPα receptor on macrophages. Pharmacological blockade and genetic perturbation of this signaling axis have shown promise as anticancer therapy^98,99^. Furthermore, it has been found that CD47 on cancer cells can interact in cis with SLAMF7 (a pro-phagocytic molecule) to evade macrophage phagocytosis^98^. In addition, a recent study identified that P-selectin glycoprotein ligand 1 (PSGL-1) is a negative regulator of phagocytosis^23^. The authors proposed a mechanism whereby PSGL-1 on tumor cells inhibited phagocytosis by blocking the tumor ICAM-1 and macrophage LFA-1 interaction to suppress pro-phagocytic downstream intracellular signaling pathways. Importantly, a PSGL-1 blocking antibody restored phagocytosis in vitro and slowed cancer progression in vivo. Together, therapeutic manipulation of macrophage phagocytosis of cancer cells has shown promising results in several in vivo models. A more comprehensive review focused on phagocytic checkpoints in the tumor immune microenvironment has been published elsewhere^100^.

The CD47-SIRPα signaling axis is a key mediator of macrophage phagocytosis. Macrophages express the transmembrane protein SIRPα, which can bind to the CD47 ligand, identifying a cell as “self” as opposed to “non-self”. When this signaling complex is formed, a “don’t eat me” signal occurs and prevents macrophage phagocytosis of the cell. Cancer cells can leverage this pathway by overexpressing CD47, thereby preventing macrophage phagocytosis^101^. Blocking the CD47-SIRPα signaling axis by using a CD47 antagonist promotes macrophage phagocytosis of cancer cells as well as biases macrophages towards an antitumor state, increasing macrophage recruitment. This has proven to be an attractive pharmacological intervention, with numerous preclinical studies demonstrating its efficacy. Accordingly, there are over 30 ongoing clinical trials primarily consisting of CD47 monoclonal antibodies or targeted bispecific antibodies to disrupt this signaling axis^102,103^. These trials are exploring solid tumors, including ovarian and breast cancers, and hematological cancers such as lymphoma, leukemia, and multiple myeloma. Several of these preclinical and clinical studies have shown promising results, but the class of drugs still faces challenges, including therapeutic efficacy. Safety has also presented itself as an issue, with many concerns stemming from the fact that many normal cells express CD47. This can lead to macrophages phagocytosing healthy cells, causing hematotoxicities (e.g., anemia). However, various strategies, including anti-SIRPα monoclonal antibodies, are seeking to overcome these limitations^102^.

Once macrophages phagocytose cancer cells, they can present tumor antigens to T cells through a process known as cross-presentation^104^. Cross-presentation involves presentation of cancer antigens on the major histocompatibility complex (MHC) I or II molecule of macrophages to the T cell receptor (TCR)-CD3 protein on T cells (called the TCR complex). MHCI and MHCII differ in both structure and immunological function. MHCI molecules are expressed on nearly all nucleated cells and present intracellular (endogenous) peptides to CD8⁺ cytotoxic T cells. In contrast, MHCII molecules are primarily expressed on professional antigen-presenting cells and present extracellular (exogenous) peptides to CD4⁺ helper T cells. Peptide MHC (pMHC) I and II ligate the TCR complex to promote anticancer functions of CD8 + T cells and CD4 + T cells, respectively^104,105^. Productive T cell activation requires pMHC-TCR complex ligation as well as the interaction between CD80/86 costimulatory molecules on macrophages and CD28 on T cells. Consequently, this stimulates adaptive immunity and provides long-term, durable responses by T cells. Importantly, the pMHC-TCR complex ligation and costimulatory molecules interaction can occur in various tissues, including the lymph node, spleen, and tumor microenvironment (TME). And the quality of the adaptive immune response might depend on the anatomical location. In the lymph node, soluble cancer antigens drain from the TME through lymphatic circulation when they can be captured by a subset of macrophages known as the lymph node subcapsular sinus macrophages^106^. The subcapsular sinus macrophages then present the captured antigens to lymphocytes (T cells or B cells) in the lymph node to activate cellular or humoral immune response, respectively. Lymph node targeting to facilitate robust communication between the innate and adaptive immunity has provided improved cancer response in multiple disease models^106–108^.

### Migration

Circulating monocytes are recruited to the TME and differentiate into macrophages when they encounter solid tissue^109^. Importantly, macrophages must migrate through tissue for them to establish proximity to cancer cells, where they can exert tumoricidal functions. As a result, the mechanisms that govern macrophage migration are an area of active investigation^90^. Macrophage migration involves interaction with ECM proteins in the TME, such as fibronectin and collagen. As a result, the role of collagen-binding receptors (e.g., β1-integrins) on macrophage adhesion to the ECM and migration has been investigated^110–112^. What has emerged from many studies is that, unlike most immune cells that only utilize amoeboid migration, macrophages utilize both amoeboid and mesenchymal migration in vivo^110,112^. Amoeboid and mesenchymal migration are characterized by distinct morphological and signaling features: the former is dependent on Rho-associated coiled-coil kinase (ROCK) and exhibits a round morphology, and the latter is ROCK-independent and exhibits an elongated morphology^113^. In addition, mesenchymal migration is often associated with Rac activity and ECM remodeling via proteolytic degradation, whereas amoeboid migration is not and involves the generation of contractile forces^114–117^. A recent study demonstrated that monocytes migrating through a viscoelastic matrix have an amoeboid-like morphology. Interestingly, these monocytes did not exhibit all the canonical traits of amoeboid migration, such as strong actin and myosin co-localization or contractile forces. Instead, actin was localized at the rear of the cell, myosin was diffuse throughout the cytoplasm, and the cells used protrusive forces to remodel the matrix to promote migration^118^. These findings suggest that cells might be able to use diverse modes of migration beyond the classical amoeboid/mesenchymal paradigm.

Chemokines and cytokines also play an important role in macrophage migration through the interstitial matrix. The CCR2-CCL2 (receptor-ligand) pair has been extensively studied and identified to play a key role in macrophage migration and subsequent accumulation in the TME^119^. Considering this, anti-CCL2 neutralizing antibodies and antisense oligonucleotides have been developed, and administration of this antibody significantly reduces monocyte/macrophage accumulation in the TME^119,120^. However, there are two points to note. First, while monocytes/macrophages express CCR2 (i.e., are CCR2 + ), the source of CCL2 has not been fully determined. Some studies have implicated CCL2 production by cancer cells as a result of the activation of the mammalian target of rapamycin complex 1 pathway, a frequently associated and mutated pathway in many cancers^121,122^. A better understanding of the non-cancer sources of CCL2 could provide additional therapeutic opportunities. Second, anti-CCL2 antibodies reduce macrophage accumulation by 50%^119^. This suggests that there are other cytokines and chemokines that recruit macrophages to the TME. In this regard, vascular endothelial growth factor and macrophage colony-stimulating factor 1 (CSF-1) have been nominated as potential candidate cytokines that support macrophage recruitment^119,120,123^.

The presence of macrophages and cellular associations correlates with tumor progression. Hence, it is important to develop a thorough understanding of the mechanisms that govern their migration and accumulation in the TME. While substantial progress has uncovered the cytokines and chemokines that govern macrophage migration, mouse models and clinical evidence suggest that the mechanisms that govern this process are not fully understood, highlighting the need for in vitro and ex vivo studies to obtain mechanistic insights^124^. It is likely that additional tissue-specific factors, including the mechanical properties of the ECM, stromal cues, tumor interstitial fluid pressure, biophysical cues, and cellular crosstalk, play key roles during in vivo macrophage migration^90^. Recent studies have quantified their trafficking and accumulation in tissues^125,126^. However, single-cell resolution of cell trafficking in vivo is still a technical barrier to understanding macrophage migration behavior under physiologically relevant conditions. Future technological advances in biological labeling, engineered macrophages, and ‘omics approaches could help to shed light on the mechanism of monocyte/macrophage recruitment during tumor progression in vivo.

### Monocyte/macrophage adhesion and inflammation

**Fig. 2:**
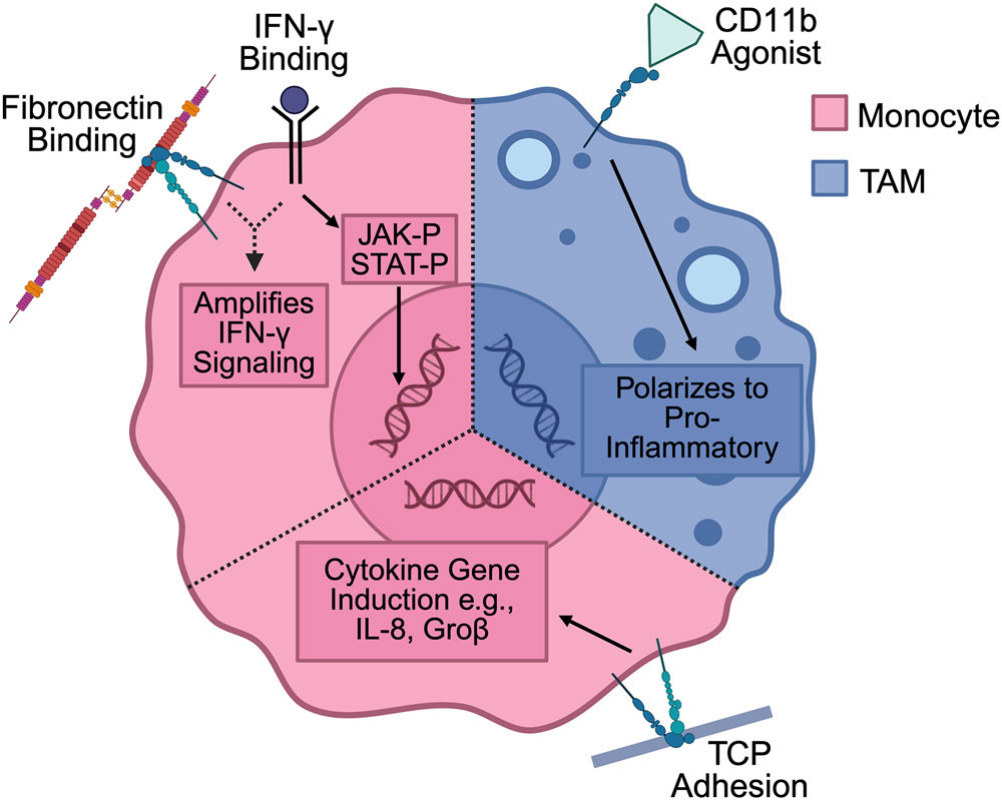
Cell adhesion and integrins modulate pro-inflammatory and cytokine signaling transduction pathways in monocytes and macrophages^131–133^. In monocytes, fibronectin-integrin binding amplifies IFN-γ signaling, particularly tyrosine phosphorylation of JAK/STAT, RNA levels for FcγR1, and presence of FcRFγ DNA-binding complex^132^. Furthermore, monocyte tissue culture plastic (TCP) adhesion induces the transcription of different cytokine genes, such as those for IL-8 and GRO-β^131^. In Tumor-Associated Macrophages (TAMs), CD11b agonist (leukadherin-1) promotes pro-inflammatory gene expression, particularly via the stimulation of Let7a and inhibition of Pdgfb and Il6^133^. Created in BioRender. Zhang, D. (2025) https://BioRender.com/prxfh42.

Acute inflammation is a normal physiological response to microbial infection or sterile tissue damage and is required to maintain tissue homeostasis^127^. In addition, it is characterized by a transient inflammatory response phase, followed by a resolution phase that is typically initiated within a few hours post-inflammation^128^. Chronic inflammation, on the other hand, results in persistent, unresolved inflammation and is associated with chronic diseases such as Alzheimer’s disease, atherosclerosis, cardiovascular diseases, and cancer^127^. Previous research has defined the biochemical cues (cytokines, chemokines, resolvins, etc.) that promote or resolve inflammation^127^. However, mounting evidence suggests that ECM proteins and macrophage-ECM (or substrate) interactions may exert an immunomodulatory effect on immune cells^129,130^ (Fig. 2). This is important because excessive ECM deposition (fibrosis) is a hallmark of many diseases, including inflammatory bowel diseases and cancer, suggesting that the cell-ECM interaction might play an important role in disease progression. Uncovering the crosstalk between cell-ECM interactions and inflammation could pave the way for the discovery of new therapies with broad applications. Accordingly, efforts are underway to identify the mechanisms responsible. For example, early work showed that the engagement of β1-integrins led to the signal induction of key cytokine genes in monocytes and monocyte-derived macrophages^131^. In addition, a separate study found that integrin-mediated binding of human monocytes to fibronectin amplifies the IFN-γ cytokine-induced signal transduction pathway, and subsequent JAK/STAT tyrosine phosphorylation^132^. It is thought that the mechanisms by which this happens are due to β1-integrins clustering or redistribution of IFN-γ receptors within the plasma membrane to throttle the level of cytokine signaling. Furthermore, a recent study suggested that integrin CD11b (α_M_ subunit)-mediated adhesion might repolarize tumor-associated macrophages to a pro-inflammatory phenotype and a subsequent robust anti-tumor response in vivo^133^. Together, these studies suggest that monocyte/macrophage adhesion to the ECM could potentiate a pro-inflammatory response, raising the possibility of leveraging macrophage-ECM interactions for therapeutic impact.

While these early studies focused on adhesion, mounting evidence suggests that macrophage adhesion and mechanobiology (see “Macrophage mechanobiology” section) are closely intertwined^89^. For example, recent evidence has uncovered crosstalk between the mechanosensitive ion channel, Piezo1, and integrins, canonical mediators of cell adhesions^134^. Interestingly, some studies have shown that Piezo1 regulates matrix adhesions to promote cell spreading in cancer cells and clustering of dorsal root ganglion neurons^135,136^. Future work will help clarify the mechanisms by which macrophages integrate mechanosensitive and adhesion signaling pathways to tune macrophage behaviors in the tumor microenvironment. In the meantime, there is a wide interest in using anti-adhesion therapies to treat acute and chronic inflammation. However, the mechanisms by which these therapies might work have not been fully understood. The studies described here provide a possible explanation for the efficacy of some anti-adhesion therapies in mediating inflammation. For example, some have argued that the mechanism of action of adhesion-disrupting peptides in the mouse arthritic model might be related to the ability of the peptides to attenuate adhesion-mediated cytokine pathways^132^. Together, these findings implicate a close link between integrin ligation, adhesion, and cytokine signaling in immune cells, particularly monocytes/macrophages. This is exciting because it suggests that the integrin-adhesion mechanotransductive pathway in non-immune cells might also be relevant to immune cells. More importantly, these observations regarding the role of the ECM in inflammation advance our understanding of the biology of acute and chronic inflammation and pave the way to develop novel approaches to rationally modulate immune cell adhesion for therapeutic impact in cancer.

### Macrophage-derived extracellular vesicles in phenotypic regulation

Macrophage-derived extracellular vesicles (EVs) have emerged as important autocrine and paracrine regulators of macrophage behavior. EVs are small vesicles released by cells that can enable macrophage populations to coordinate inflammatory and reparative responses. These cellular products are diverse and vary in size ( ~ nm to μm), composition, and molecular content (proteins, mRNAs, miRNAs, DNA), which can sculpt the tumor immune microenvironment^137–139^. For example, TAMs EVs have been found to be enriched in miR-21-5p and miR29a-3p. The molecules promote an immunosuppressive tumor microenvironment by inhibiting the STAT3 pathway and induction of Treg/Th17 cell imbalance, ultimately facilitating the progression and metastasis of epithelial ovarian cancer^137,140^. Interestingly, a separate study found that macrophage-derived EVs might have an anti-tumor immunity in MC38 colorectal tumors^139^. The opposing findings suggest that EVs might play diverse roles depending on the tissue and disease context. Together, these findings indicate that macrophage-derived EVs can act as a powerful mechanism of cell-cell communication that enables macrophages to modulate their functional behaviors and the local microenvironment within tissues.

## Macrophages: from bench to bedside

The role of the immune system in responding to cancer has been debated over time^141^. Early work observed that cancerous tissues often contained immune cells, suggesting that immune cells might be involved in cancer progression^142,143^. Additional evidence that implied relevance of our immune system to treat cancer was published in the late nineteenth century with the use of “Coley’s Toxins”^144,145^. Recently, powerful evidence has emerged that strongly implicates the immune system in cancer. For example, many studies have demonstrated the prognostic value of tumor-infiltrating immune cells across several human cancers, including colorectal, melanoma, breast, and liver cancer^146–149^. We now know that macrophages exhibit tumoricidal functions, directly implicating the immune system in tumor control^150,151^. Macrophages in particular are effectively recruited to the solid tumor microenvironment^152^. Once in the TME, macrophages can adopt a range of classic phenotypes whose extremes range from a proinflammatory to immunosuppressive^15,153,154^. Specifically, TAMs have been extensively studied due to their diagnostic, prognostic, and therapeutic potential^155,156^. Recent advances have revealed substantial heterogeneity in the TAM population^16,157–159^. This heterogeneity is also associated with diverse functions in the TME^16^. The ability of macrophages to infiltrate and phagocytose tumors and present tumor antigens to T cells makes them attractive therapeutic targets to serve as a bridge between innate and adaptive immunity^109^. Accordingly, several therapeutic approaches have emerged to leverage these intrinsic functions of macrophages in the TME. These approaches can broadly be classified as genetic or non-genetic engineering of macrophages and are described below.

### Genetic engineering of macrophages

**Fig. 3:**
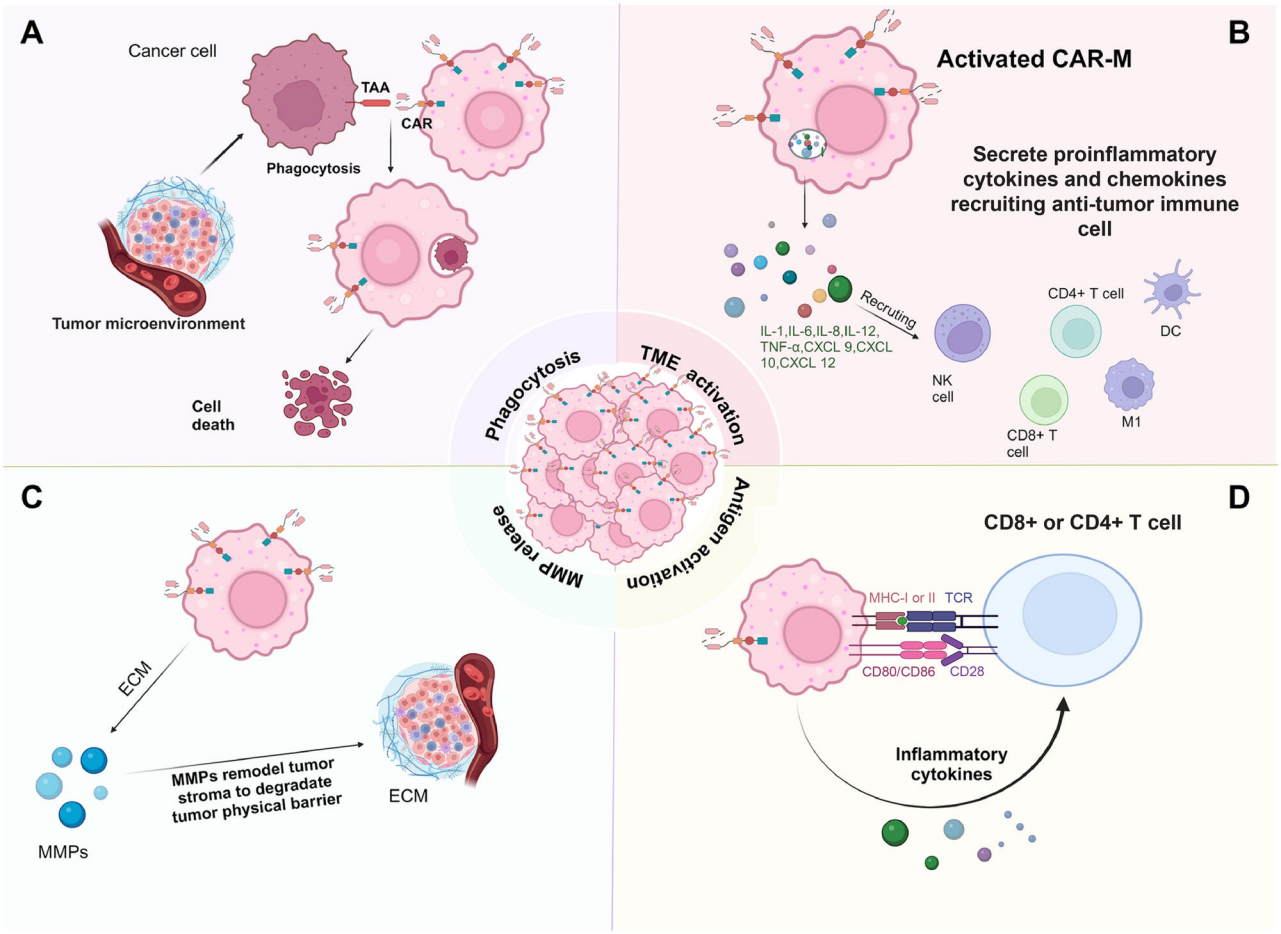
Major mechanisms for CAR-Ms to clear solid tumors. **A** CAR-Ms can engulf and kill tumor cells, which are recognized by CAR through tumor cell-associated antigens. **B** Activated CAR-Ms can secrete proinflammatory cytokines and chemokines to recruit anti-tumor immune cells to the tumor microenvironment. **C** CAR-Ms can induce the secretion of matrix metalloproteinases to degrade the dense tumor ECM. **D** CAR-Ms can activate CD8^+^ or CD4^+^ T cells to become anti-tumor by cross-presenting antigens from phagocytosed cells via MHC-I or II, expressing co-stimulatory ligands CD80 or CD86, and secreting inflammatory cytokines.TAA: tumor-associated antigen, MMP: matrix metalloproteinase, TCR: T cell receptor. Adapted from Yang, S. et al.^224^. Licensed under CC BY 4.0. This is an open-access article distributed under the terms of the Creative Commons CC BY license, which permits unrestricted use, distribution, and reproduction in any medium, provided the original work is properly cited. Figure taken from the original paper with minor modifications made in Adobe Illustrator ® to improve legibility. Engineered approaches to macrophage cell therapies https://creativecommons.org/licenses/by/4.0/.

Chimeric antigen receptor (CAR) T cell therapies have revolutionized the treatment of hematological cancers but show limited efficacy against solid tumors. CARs are synthetic, modular receptors composed of an extracellular antigen-binding domain, typically a single-chain variable fragment, linked to a transmembrane region and intracellular signaling motifs. This architecture enables a cell of interest (e.g., T cells, NK cells, or macrophages) to recognize antigens independently of MHC and initiate downstream activation programs. The modular design allows each component to be tuned for affinity, signaling strength, and cellular response. It is thought that poor CAR-T tumor infiltration could be a contributor to the limited efficacy^160–162^. Macrophages, however, can actively and effectively infiltrate tumors^163,164^. Therefore, it has been suggested that the introduction of the CAR into macrophages (CAR-M) could take advantage of the tumor-homing functions of macrophages while endowing them with cancer-fighting properties against poorly immunogenic or heterogeneous tumor antigens. In addition, CAR-M’s could broaden antigen recognition through cancer phagocytosis and subsequent tumor antigen presentation. But it is known that macrophages are notably resistant to genetic modification using conventional vectors like lentiviruses, retroviruses, and adeno-associated viruses. However, several groups have succeeded in making CAR-Ms (or genetically engineered macrophages, GEMs) using modified viruses^165–167^. One strategy that has been employed to genetically modify macrophages is transduction of the CAR with adenoviral vectors such as Ad5f35. This system relies on the endogenous expression of CD46 on macrophages, which serves as the natural docking receptor for group B adenoviruses^168^. Several iterations of CAR-Ms have been developed^169^. Armed with a CAR, macrophages are then poised to leverage the unique properties of macrophages, such as cancer phagocytosis, antigen presentation to T cells, and tumor trafficking. Early work with CAR-Ms sought to promote cancer phagocytosis. Chimeric Antigen Receptors for Phagocytosis (CAR-Ps) were combined with cytoplasmic domains capable of promoting phagocytosis, such as Megf10 (multiple EGF-like-domains protein 10)^170^. Phagocytosis was further enhanced by including the Phosphoinositide 3-kinase (PI3K) signaling motifs that are known to promote phagocytosis^171^. More recent work demonstrated enhanced phagocytosis of opsonized tumor organoids^95^. Another study showed that macrophage clustering is a key biophysical mechanism that promotes tumor phagocytosis^172^. Macrophages will likely have to act in concert with other immune cells to promote a robust anti-tumor response. Hence, there have been intense efforts to promote macrophage antigen presentation to potentiate T cell activation^173^. This crosstalk between macrophages and effector T cells is important to combat the various tumor mechanisms that suppress the immune system in cancer, such as tumor antigen heterogeneity. By sampling the tumor through phagocytosis and global upregulation of the antigen processing machinery in the TME, macrophages have the potential to present a holistic view of the tumor biology to the adaptive immune system (Fig. 3)^168,173^.

Overall, the CAR-M is a promising technology and has proceeded to human clinical trials (Table 1) where it has shown a good safety profile, no dose-limiting toxicity, and severe cytokine release syndrome has not been reported. The NCT04660929 trial is a landmark first-in-human study of CAR-macrophages (CT-0508) targeting patients with advanced HER2-overexpressing tumors. Preliminary results are encouraging and demonstrate preliminary safety, manufacturability of therapeutic doses, and biological activity^168^. But the efficacy data for this trial is preliminary, and further optimization of patient selection, dosing regimen, dose escalation, and combination treatments might be required to maximize clinical efficacy. This trial represents pioneering work in CAR-macrophage (CAR-M) therapy, especially for solid tumors, where conventional CAR-T faces major obstacles (like poor infiltration or an immunosuppressive microenvironment. Other trials have either been suspended with no results publicly posted (NCT05138458) or there is limited public information available regarding clinical efficacy or safety outcomes (NCT06562647, ChiCTR2400080078), and two trials are not yet recruiting (NCT06224738, ChiCTR2400082776).

**Table 1:** Clinical trials investigating CAR-M treatments

ID	Country	Start year	Status	Phase	Indication	Macrophage source
NCT04660929	USA	2021	Active, not recruiting	1	HER2 overexpressing solid tumors	Autologous monocytes
NCT05138458	USA	2021	Suspended	1/2	T Cell Lymphoma	Autologous myeloid cells
NCT06562647	China	2023	Recruiting	N/A	Mesothelin (MSLN) overexpressing solid tumors	Autologous PBMCs
ChiCTR2400080078	China	2024	Recruiting	1	Relapsed/refractory ovarian cancer with High HER2 expression	N/A
NCT06224738	China	2024	Not yet recruiting	Early 1	HER2+ advanced gastric cancer with peritoneal metastases	Autologous bone marrow stem cells
ChiCTR2400082776	China	2024	Not yet recruiting	N/A	Solid tumors	N/A

The NCT trials are from www.clinicaltrials.gov. The ChiCTR trials are from https://www.chictr.org.cn/searchprojEN.html.

### Non-genetic, materials-based engineering of macrophages

**Fig. 4:**
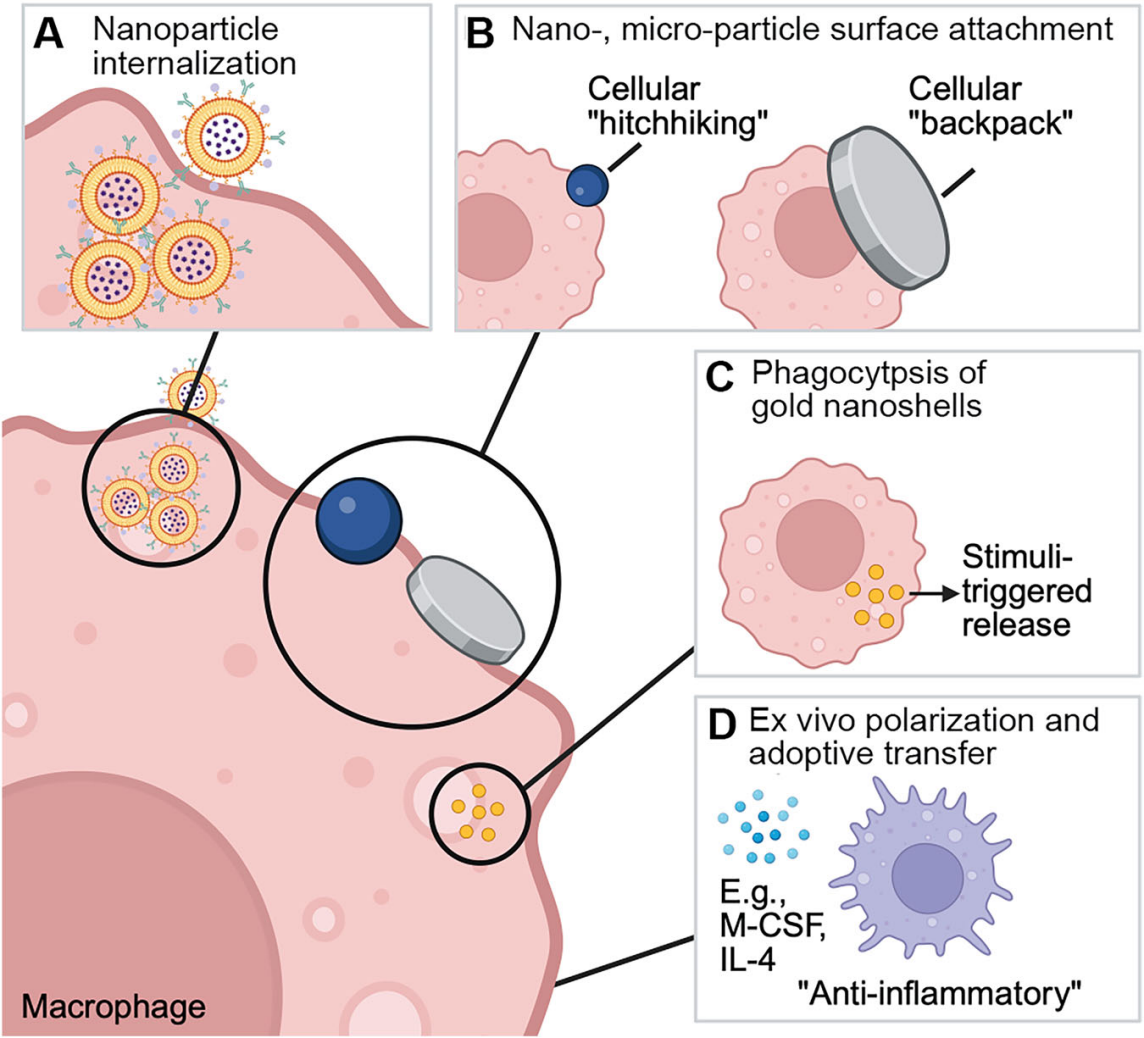
Engineered approaches to macrophage cell therapies. **A** Nanoparticles containing a therapeutic drug are phagocytosed by the macrophage. The macrophage then migrates to a tumor site and delivers the drug cargo. **B** Nanoparticles (“hitchhiking”) or microparticles (“backpacks”) are attached to the macrophage cell surface, with or without cargo, to maintain desired phenotypes. **C** Gold nanoshells are phagocytosed by macrophages and then released into the tumor microenvironment via photoablation. **D** Macrophages are polarized ex vivo with an activation cocktail, allowing for a sustained anti-inflammatory phenotype when reintroduced. Adapted from Na et al.^225^. Licensed under CC BY 4.0. Created in BioRender. Adebowale, K (2025) https://BioRender.com/iju20xa.

Non-genetic approaches to engineer macrophages have focused on using materials to modulate the behavior of macrophages. These materials include liposomes, dendrimers, nanoparticles, and microparticles that usually contain a therapeutic payload (Fig. 4)^174–183^. This materials-based approach offers greater flexibility and less complex ex vivo manipulation, leading to potentially greater ease of implementation. Many of these strategies also leverage endogenous macrophage functions such as tissue infiltration, phagocytosis, and phenotypic plasticity. Initial efforts in this area focused on macrophages’ phagocytic ability and ease of infiltrating tissues, utilizing them as advanced drug delivery vehicles. With this approach, macrophages are incubated with nanoparticles containing a drug such as paclitaxel^34^. Macrophages then phagocytose the nanoparticle and can then migrate towards tumors to deliver the therapeutic cargo when injected into mice using a “Trojan horse” approach^34,184,185^. In one example, macrophages phagocytosed gold nanoshells over a 24-hour incubation period. Photoablation was then used to induce cell death in macrophages containing nanoshells, leading to the release of nanoshells within the tumor microenvironment. This enables their uptake by tumor cells, followed by near-infrared (NIR) irradiation to heat and destroy the tumor tissue. Electroporation, rather than direct incubation, could be a more effective method for delivering therapeutic cargo that can be easily degraded, like nucleic acids or enzyme precursors^34^. In addition, preclinical studies have shown promising results that small molecules can be used to modulate macrophage phenotype, further expanding the scope of targeted approaches to modulate macrophage phenotypes^180,186^. Small-molecule CSF1R inhibitors such as PLX3397 (pexidartinib) and BLZ945 have entered clinical development, but as monotherapies, they showed only limited efficacy, likely due to compensatory immunosuppressive pathways^187^.

Internalized therapeutic cargo could negatively impact the endogenous function of macrophages or the efficacy of the therapeutic cargo. To overcome this, therapeutic cargoes can be attached to the surface of macrophages using a “backpack” or “hitchhiking” approach^188^. Here, a payload hitchhikes on the macrophage cell membrane and is delivered at a target site. In another example, biodegradable microparticles (“backpacks”) containing IFN-γ cytokine were attached to the surface of macrophages to maintain an anti-tumor phenotype in vivo^177^. It is possible that these “backpacks” can be loaded with cargoes of interest, including small molecules, biologics, or even imaging agents. Indeed, these “backpacks” were recently loaded with gadolinium contrast agent to monitor macrophage trafficking in the brain^189^. Such an approach could help provide quantitative information about the in vivo cellular kinetics of adoptively transferred macrophages, thereby facilitating the development of predictive models.

Macrophage-derived extracellular vesicles are being investigated as cancer treatments and have been shown to exert anti-tumor effects in pre-clinical models. These extracellular vesicles are typically derived from mouse or human cell lines (RAW 264.7 or THP-1), primary mouse macrophages (bone marrow-derived macrophages, peritoneal-derived macrophages, adipose tissue-derived macrophages), or monocytes isolated from human peripheral blood^190^. These extracellular vesicles typically contain microRNAs with known immunomodulatory functions or chemotherapeutic drugs and have been described in recent reviews^137,190^. Because extracellular vesicles are cell-derived, it is thought that they will eventually have a good safety profile, low immunogenicity, good biocompatibility, and low toxicity in humans. They could also be modified ex vivo to direct their uptake in tissues of interest. However, the majority of clinical trials on extracellular vesicles focus on cancer prognosis or diagnosis, while only a few focus on treatment^191^. This suggests that therapeutic applications in humans are still in the early stages. Some of the challenges that need to be overcome include optimizing cargo loading and efficiency, producing homogenous vesicles with tailored characteristics that achieve precise in vivo targeting to provide improved therapeutic potential. Despite these challenges, progress in this field is progressing at a rapid pace that could accelerate the clinical impact of extracellular vesicles on patient outcomes.

## Conclusion and perspectives

Macrophages play dual roles in cancer by promoting or suppressing disease progression. Accordingly, researchers have developed two main strategies to inhibit their pro-tumor or enhance their anti-tumor role. Some strategies have focused on inhibiting the recruitment of macrophages into tumors and have resulted in clinical trials^192^. Most of these clinical trials target the CSF1R and CCR2 signaling pathways (receptors for CSF-1 and MCP-1, respectively) or are used in combination with other cancer therapies^192,193^. Other strategies have sought to leverage the endogenous tumor-homing properties of macrophages, while endowing them with genetic or non-genetic modifications to prevent them from reverting to their pro-tumor phenotypes^163^. Macrophage cell therapies are alive, polyfunctional, heterogeneous, and often have many mechanisms of action; therefore, their physicochemical and pharmacological properties are different from small molecules of biologics^194^. Hence, it is important to define their cellular kinetics and pharmacodynamics^194,195^. Magnetic resonance imaging can help quantify macrophage accumulation in tissues, but advances in imaging will be needed to obtain greater spatiotemporal resolution^125,126^.

Macrophage functions are more complex than the M1-like/M2-like binary classification. Powerful evidence to support this has emerged from rapid advances in single-cell sequencing, spatial transcriptomics, and transgenic in vivo models. Macrophages likely exist on a phenotypic spectrum, constantly changing their functions in response to the changing local biochemical milieu. Of note, recent evidence has emerged that macrophages are mechanosensitive cells that sense and respond to the biophysical and mechanical properties of tissues and cells they interact with^35,36,70,75,76,89,196–202^. Thus, there is a need to re-evaluate macrophage behaviors through the framework of mechanobiology. In this regard, researchers can use bioengineered systems to study macrophage behaviors under controlled, but physiologically relevant, conditions. We envision that these bioengineered systems can leverage advances in 3D cell culture, matrix engineering, cell surface engineering, nanoengineering, bioprinting, organoids, micro-physiological systems, and organs-on-a-chip technologies to mimic physiologically relevant mechanical and biophysical cues^203–219^. Moreover, results from these experimental platforms can help to rigorously quantify the cellular kinetics and pharmacodynamics of cell therapies. Furthermore, the results can be combined with ‘omics data, computational modeling, and machine learning approaches to promote a deeper understanding of macrophage behaviors. Such an integrated, interdisciplinary approach can accelerate our efforts to establish the fundamental principles that govern macrophage behaviors, thereby facilitating our ability to predict their in vivo behavior.

T cell exclusion from solid tumors remains a therapeutic challenge in immuno-oncology. However, many tumors with a T cell-excluded signature (immunologically cold) have lots of macrophages. Thus, macrophages might be a promising candidate under such scenarios where T cells are excluded from the tumor nest. In this regard, a recent study demonstrated that macrophages transfer drugs directly to an immunologically cold glioblastoma cancer cells upon physical contact^193^. These results are encouraging for this difficult-to-treat tumor with a median survival of 14 to 15 months after diagnosis^220^. Furthermore, CAR-M therapy also demonstrated anti-tumor efficacy in the poorly immunogenic 4T1 pre-clinical model of breast cancer in mice^221^. While there was reduced tumor burden, suggesting an anti-tumor effect, there was no improvement in survival. Nevertheless, these results demonstrate the potential of macrophage therapies to treat immunologically cold cancers.

While macrophages are promising as a cell therapy, several challenges remain that could limit their clinical impact. One challenge involves the ex vivo differentiation of macrophages from circulating monocytes, which can involve considerable cell loss, is lengthy, and also expensive^222^. This limitation could be partially addressed by using allogeneic iPSC-derived macrophages instead of circulating monocytes as the cell source. The use of allogeneic cells also supports the feasibility of an off-the-shelf approach and could make this therapy widely accessible. An intriguing solution to avoid cell loss is in vivo mRNA CAR-M cell engineering from monocytes, as was recently demonstrated for CAR-T engineering^223^. Furthermore, the phenotypic plasticity of macrophages might make it difficult to achieve a reliable and consistent therapeutic product in the complex tumor microenvironment. Here, engineering approaches and deeper insights into macrophage behaviors might facilitate strategies to maintain desired phenotypes. Another challenge with macrophage therapies is that the cells have limited proliferative potential. On the one hand, this is beneficial because it reduces the risk of long-term complications. On the other hand, this could reduce the long-term persistence of therapeutic macrophages in vivo within the tumor. In fact, recent studies suggest that CAR-Ms might have limited trafficking potential in humans^168^. Accordingly, it will be beneficial to develop and utilize bioengineered systems that investigate the factors that contribute to limited trafficking and a short half-life in tumors. In this regard, monocytes can be a case study to investigate because, compared to macrophages, they have superior trafficking behavior and their half-life in mice is thought to be tenfold higher compared to macrophages^124,168^.

In conclusion, engineered therapeutic macrophages are a promising therapy to treat cancer. Macrophages possess endogenous functions that make them suitable for this task, including tissue trafficking, tumor phagocytosis, and activation of adaptive immunity. Bioengineered systems will help to further define the biochemical, mechanical, and biophysical cues that regulate these functions. Therapeutic macrophages might also be able to effectively navigate the regulatory landscape. This is because human data suggest that adoptive macrophage cell therapies have a good safety profile and might have a reduced risk of long-term immune complications. In sum, macrophage therapies offer new hope for solid, difficult-to-treat tumors that are often characterized by immunosuppressive and treatment-resistant niches.

## Data Availability

No datasets were generated or analyzed during the current study.
